# A twin approach to unraveling epigenetics

**DOI:** 10.1016/j.tig.2010.12.005

**Published:** 2011-03

**Authors:** Jordana T. Bell, Tim D. Spector

**Affiliations:** 1 Department of Twin Research and Genetic Epidemiology, King's College London, St. Thomas’ Hospital, Westminster Bridge Road, London SE1 7EH, UK; 2 Wellcome Trust Centre for Human Genetics, University of Oxford, Roosevelt Drive, Oxford OX3 7BN, UK

## Abstract

The regulation of gene expression plays a pivotal role in complex phenotypes, and epigenetic mechanisms such as DNA methylation are essential to this process. The availability of next-generation sequencing technologies allows us to study epigenetic variation at an unprecedented level of resolution. Even so, our understanding of the underlying sources of epigenetic variability remains limited. Twin studies have played an essential role in estimating phenotypic heritability, and these now offer an opportunity to study epigenetic variation as a dynamic quantitative trait. High monozygotic twin discordance rates for common diseases suggest that unexplained environmental or epigenetic factors could be involved. Recent genome-wide epigenetic studies in disease-discordant monozygotic twins emphasize the power of this design to successfully identify epigenetic changes associated with complex traits. We describe how large-scale epigenetic studies of twins can improve our understanding of how genetic, environmental and stochastic factors impact upon epigenetics, and how such studies can provide a comprehensive understanding of how epigenetic variation affects complex traits.

## Epigenetic mechanisms

The term epigenetics was originally introduced to describe how interactions between genetics and environment can give rise to phenotypes during development [1]. Epigenetics today more specifically defines cellular modifications that can be heritable, but appear unrelated to DNA sequence changes, and can be modified by environmental stimuli [2,3]. At present, epigenetic mechanisms typically comprise DNA methylation and histone modifications, but also include many other mechanisms such as ATP-based chromatin-remodeling complexes, Polycomb–Trithorax protein complexes, non-coding RNA mediated gene-silencing, and potentially prions, transcription-factor binding, and other mechanisms involved in generating and maintaining heritable chromatin structure and attachment to the nuclear matrix. Epigenetic mechanisms play an essential functional role in complex organisms as regulators of transcription. Central to epigenetic regulation is the modulation of chromatin structure, whereby the majority of epigenetic processes impact upon chromatin organization and maintenance. Next-generation sequencing technologies have been developed to assay epigenetic changes (Box 1) in high-throughput approaches, and high-resolution genome-wide epigenetic profiles promise a more complete understanding of the functional impact of epigenetics. Of these processes, DNA methylation is the mechanism that has been studied in the greatest depth, and we therefore focus predominantly on this mechanism in this review.

Epigenetic mechanisms are present in many taxa, but DNA methylation has been most extensively studied in mammals where it appears to be restricted to the cytosine base, and especially in the context of CpG dinucleotides. CpG dinucleotides are cytosine–phosphate–guanine sequences that typically cluster in genomic regions referred to as CpG islands (CGI), which are often located in gene promoters and exhibit low levels of DNA methylation. However, DNA methylation in mammals can also occur outside the CpG context, and this has been reported for example in embryonic stem cells [4]. Furthermore, although cytosine is typically methylated to 5-methylcytosine it can also be converted to 5-hydroxymethylcytosine, which could also play an important epigenetic role [5]. In mammals, DNA methylation is mediated by DNA methyltransferases that are responsible for *de novo* methylation and the maintenance of methylation patterns during replication [6], and also by DNA demethylases that remain largely unknown. There are several assays for genome-wide evaluation of DNA methylation patterns (Box 1), and methylation cross-technology comparisons have shown high concordance between different sequence-based methods [7] and slightly lower concordance between sequenced-based and microarray-based methods [8,9].

**Figure 1:**
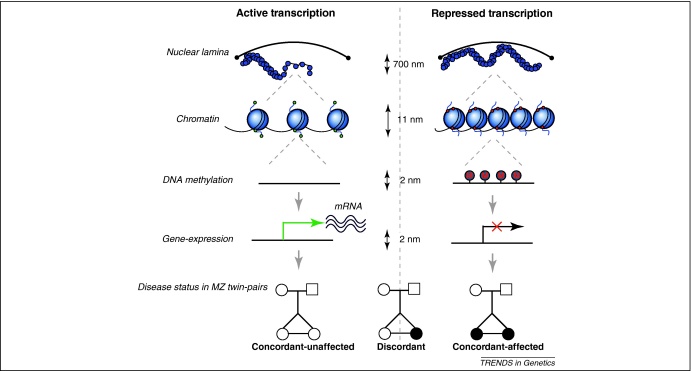
Epigenetic changes and their effects on transcription and disease. Epigenetic variants across multiple levels of chromatin structure, shown here at different levels of cell resolution in nanometers (nm), associate with gene expression and disease status in a sample of MZ twins. Top, higher-order chromatin loop configurations and attachment to the nuclear lamina can represent active and repressed chromatin domains that associate with differential gene expression. The next level represents the chromatin ‘beads on a string’ configuration, which reflects structural organization into loosely structured (active) and densely packed (repressed) chromatin states. Histone modifications associated with active transcription (green) and transcription silencing (red) are indicated by colored dots. The next levels of cell resolution depict DNA methylation (red M) in the promoter regions of the silenced genes and the corresponding differences in gene expression. Bottom, possible effects of these changes on disease status in a sample of MZ twins, highlighting unaffected-concordant, discordant, and disease-concordant MZ twins.

Cytosine methylation is essential in mammalian development, particularly in cell-lineage specification [10–12], in the regulation of transcription [13–18], and in maintaining genome stability [10,16,19]. Correspondingly, variable DNA methylation patterns mirroring the functional context of genomic regions have been observed in regulatory regions, in promoters and gene-body regions, and in repetitive elements [10,19–23], suggesting that different mechanisms could be involved in the regulation of DNA methyltransferase activity across the genome and in the interaction with chromatin-associated proteins and histone modifications [24,25]. Discrete changes in cytosine methylation at CpG dinucleotides in gene promoters can induce stable silencing of gene expression both in normal development [10] and in disease [26]. Overall, patterns of negative correlation between promoter methylation and gene expression have been observed across multiple organisms and tissues [20–22,27]. Furthermore, such negative correlations are more striking in CGI shores, defined as regions up to 2 kb outside of CGI borders, suggesting a functional role for these genomic regions in tissue differentiation and disease [27,28]. In addition, in genomic imprinting only one parent-of-origin copy of the gene is expressed, and the other is silenced via differential DNA methylation. For example, differentially methylated regions (DMRs) at the human *H19* locus control imprinting and gene expression at the maternally imprinted and transcriptionally-silenced insulin-like growth factor II (*IGF2*) locus and at the paternally imprinted and silenced *H19* region [29,30]. DNA methylation is also strongly correlated with other epigenetic changes, especially histone modifications, implicating shared mechanisms of epigenetic regulation and downstream effects [13,25,31]. The possibility that transcriptionally silent chromatin could be a target for *de novo* DNA methylation has also been suggested (see [32]). Altogether, multiple factors including DNA sequence, DNA methylation, histone modifications, and other epigenetic and transcriptional activity factors contribute to chromatin regulation, which in turn modulates transcription and affects mammalian development and disease (Figure 1).

Studies of twins have been crucial to disentangling the contribution of genetic factors to numerous complex traits. Twin studies in epigenetics have the potential to address two important questions. First, to what extent are epigenetic changes heritable and how much variation is there in epigenetic heritability across the genome? Comparisons within and between twin-pairs can help to determine the extent of epigenetic heritability and stability. Second, do epigenetic factors contribute to complex phenotypes? Monozygotic (MZ) twins are traditionally regarded as genetically identical, therefore any phenotypic differences within MZ twin pairs are classically attributed to environmental factors. However, epigenetic variants can also associate with phenotypic differences, and the identification and interpretation of such associations is currently an important area of research. Epigenetic studies of disease-discordant MZ twins, who are completely matched for genetics, age, sex, cohort effects, maternal influences and common environment, and are closely matched for other environmental factors, should be considerably more powerful in detecting disease-related epigenetic differences than epigenetic studies of unrelated disease cases and controls with different life-histories. In the following sections we consider the value of twin studies in epigenetics and discuss recent findings highlighting the possibility that epigenetic variation can be transmitted through generations and impact upon common diseases.

## Epigenetic heritability

Heritability is the proportion of the phenotypic variance in the population that is attributed to genetic variation. Heritability estimates are traditionally obtained by comparing the extent of similarity between relatives in classical twin studies, twin-adoption studies, sib/half-sib studies, and transgenerational family studies. Each has weaknesses, but for most traits twin studies are generally regarded as the most reliable because they are unbiased by age effects and offer the ability to separate common environment from genetic effects [33,34]. In twins, heritability estimates compare concordance rates or intra-class correlations in monozygotic (MZ) and dizygotic (DZ) twins (Box 2). Twin comparisons of genome-wide epigenetic profiles can determine whether particular regions of the genome have higher epigenetic heritability estimates. In such regions, DNA methylation would appear to be influenced by genetic variation and DNA methylation variants would be relatively stable and could associate with genetic variants.

Several studies have examined DNA methylation patterns in twins. Early work focused on X-chromosome inactivation patterns in females – where one X chromosome is inactivated at random and the silent state of the inactive X-chromosome is maintained by DNA methylation [35]. The results indicated that skewed X-chromosome inactivation patterns are more frequent with increasing age and that underlying heritable patterns are present in this supposedly random process [36]. Subsequent studies focused on DNA methylation variability and heritability, and its relationship to age. Initial findings, based either on methylation assays at a few genomic regions in a moderate sample, or on methylation assays at multiple genomic regions in a small sample, indicated that epigenetic variation at specific genomic regions can be heritable, but can also change over time. The first large-scale study examined DNA methylation and histone acetylation at multiple genomic regions in 20 3-year-old and 20 50-year-old Spanish MZ twin pairs [37] and observed that MZ twins have very similar epigenetic profiles, indicative of high epigenetic heritability. However, epigenetic variability increased with age across multiple tissues and, interestingly, the greatest differences were observed *post hoc* in twins who differed most in lifestyle. However, the study was cross-sectional instead of longitudinal, potentially obscuring comparison of individual variability; furthermore, comparing epigenetic patterns in fast-growing children and adults might not be generalizable to the aging process. To this end, a recent longitudinal study of twin epigenetic heritability assayed DNA methylation patterns in the promoters of three genes in 46 MZ and 45 DZ twin pairs sampled at 5 and 10 years of age [38]. One gene showed evidence for heritability, whereas methylation differences were present in all genes at an early age and increased with time. Time-related changes in methylation have been further identified as age-related locus-specific variation in methylation across multiple tissues in samples of singletons and twins [39–41].

Substantive evidence for epigenetic heritability has been obtained from further studies of age-matched twins using larger samples [42,43] or higher-resolution DNA methylation assays [44]. Two locus-specific studies assayed DNA methylation in the 11p15.5 genomic region surrounding the maternally imprinted insulin-like growth factor II (*IGF2*) gene and the paternally imprinted *H19* region, estimating high epigenetic heritability in those regions using multiple tissues from 182 newborn MZ and DZ twins [43] and whole blood from 196 adolescent and 176 middle-aged MZ and DZ twins [42]. The most recent large-scale twin study of DNA methylation [44] used a high-resolution DNA methylation array [45] in three tissues [buccal, gut and white blood cells (WBC)] in approximately 20 MZ twin-pairs and across two tissues (buccal and WBC) in 20 DZ age-matched twin pairs. Overall, they found that MZ twins have more similar DNA methylation patterns than DZ twins across tissues. This was greatest in buccal smears and lowest in WBCs, potentially due to cellular heterogeneity because buccal cells are a homogeneous mixture of cell types. The most heritable CpG sites were correlated with functional regions and promoters, suggesting that the more functionally-relevant methylation signals were under stronger genetic control.

Overall, these findings confirm that DNA methylation is a heritable trait on a genome-wide basis, but also highlight the importance of taking age into account when studying epigenetic processes. Furthermore, the results are consistent with recent population-based findings of quantitative trait loci (QTL) for DNA methylation [46–48], transgenerational and family clustering of methylation patterns [49,50], and heritable effects of other epigenetic processes [51,52].

## Interpreting epigenetic heritability

DNA methylation patterns can be affected by genetic variation, environmental changes, heritable and non-heritable changes in other epigenetic processes (for example, chromatin structure or transcription factor binding might influence DNA methylation patterns), and stochastic changes over time. All these factors can contribute to DNA methylation heritability estimates, which are therefore time-, tissue-, locus- and population-specific. There are three further important aspects of epigenetic heritability. First, does twin epigenetic heritability reflect stability in methylation transmission during mitosis and meiosis? Second, does epigenetic variation contribute to phenotype heritability? Finally, how do epigenetic heritability findings relate to time-dependent methylation changes?

**Figure 2:**
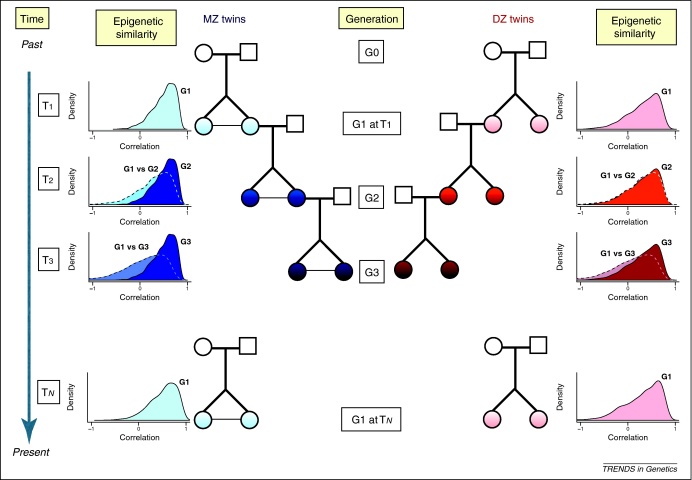
Transgenerational changes in epigenetic variation in twins. The figure provides illustrative examples of epigenetic heritability estimates in hypothetical families that include pairs of MZ or DZ twins across three generations. This highlights the idea that epigenetic heritability estimates from twin studies are expected to be higher than those obtained from transgenerational families. At each of three generation (G1, G2, G3) we represent MZ (blue) and DZ (red) intra-class correlation distributions for genome-wide DNA methylation patterns, and these are consistent with reported correlation estimates [44]. We compare within-generation correlation distributions to hypothetical transgenerational correlations in DNA methylation from parent–offspring (G1 versus G2) and grandparent–grandchild (G1 versus G3) pairs to illustrate the fact that epigenetic heritability becomes diluted over generations. In addition, the figure also emphasizes the time-specific aspect of epigenetic heritability estimates and specifically the reduction in correlation of genome-wide DNA methylation patterns for one pair of twins (at generation G1) with age, where genome-wide methylation correlations are slightly lower at later (T_N_) than at earlier (T_1_) stages in life. This is consistent with the observed increase in epigenetic variance in older twins [37,38].

In mammals, maintenance DNA methyltransferases and histone methyltransferases ensure propagation of epigenetic marks through mitotic cell divisions with high fidelity (in the range of 95–99% [53,54]) and precision [55]. However, both tissue specificity and meiotic methylation erasure argue for imperfect stability of methylation transmission. Although twin epigenetic studies are ideal for estimating the variance due to genetic factors, they could overestimate the transmissibility of these factors [56]. There is evidence that the rate of transmission of epigenetic marks lessens with each generation [57], suggesting that epigenetic profiles are likely to be more similar in families within generations as opposed to between generations (Figure 2). Whereas in plants transgenerational inheritance of DNA methylation can be relatively stable for up to eight generations [48,50], in animals meiotic epigenetic erasure should obliterate transmission of epigenetic variants. Although genome-wide data have not yet been reported, there are a few single-locus examples of transgenerational epigenetic inheritance in animals [58,59]. In general, twin studies give higher heritability estimates for common traits than do family studies, particularly in age-related diseases such as osteoporosis [60]. This could be for at least two reasons. First, it is possible that a proportion of epigenetic changes are not faithfully transmitted to offspring during meiosis, and twin epigenetic heritability overestimates the stability of meiotic methylation transmission. Second, methylation-changes accumulate with age, and therefore it is possible that age alone could explain lower family-based epigenetic heritabilities if age is not adjusted for or if age influences methylation in a non-linear manner. For example, if DNA was collected from all family members at birth, then twin-based and family-based epigenetic heritability estimates could be much more similar.

The underlying question is to distinguish between cases where methylation is completely determined by the genotype and meiotic transmission is very stable, and cases where meiotic transmission is not stable or genotype does not affect methylation [55]. Comparisons of epigenetic heritability estimates from twins and multigenerational family studies should establish whether twin heritability directly relates to the stability of meiotic transmission. Epigenetic twin heritability estimates, transgenerational studies, and DNA methylation QTL studies suggest that both scenarios are plausible, but a better understanding of the mechanisms underlying meiotic transmission, maintenance methylation, and *de novo* methylation is required.

**Figure 3:**
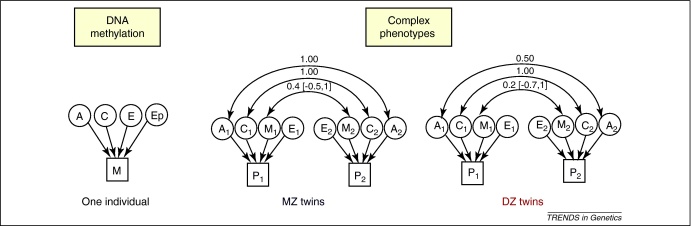
Epigenetic and phenotypic heritability. Path diagrams detailing the proposed contribution of latent variables to the methylation status of an individual at one genomic region (M) and to their phenotype (P). The left panel represents latent variables contributing to DNA methylation status at one genomic region in one individual: effects will be specific to the age, sex, and population of the individual and the tissue sampled. Methylation latent factors include additive genetic factors (A), common environmental factors (C), unique environment (E), and heritable and stable epigenetic factors that are not DNA-sequence dependent (Ep). The right panel represents the path model in twins, depicting the contribution of DNA methylation and other factors to the phenotype (P) in twin *i* with correlation estimates in MZ (left) and DZ (right) twins for latent variables including additive genetic effects (A_i_), common environment (C_i_), DNA methylation (M_i_) and unique environment (E_i_). Correlation estimates were obtained from previous genetic [114] and epigenetic studies [44] in twins. In siblings, the correlation in M will probably be lower than that observed in DZ twins due to age differences and a higher proportion of stochastic changes.

Epigenetic changes clearly contribute to phenotypes, but the extent to which they contribute to phenotype heritability is unknown (Figure 3). Hence, whether epigenetic changes explain part of the missing heritability of genome-wide association (GWA) studies [61,62] also remains far from clear. The missing heritability refers to the paradox that GWA studies have identified many genetic variants associated with complex human diseases and traits, but most variants explain only a small proportion of familial clustering. To address this point with respect to epigenetics, a notable recent study [63] extended the genetic model of Risch [64–66] which relates genetic and phenotypic variation in a mathematical framework. Slatkin [63] included both epigenetic and genetic factors in a single model to estimate their joint contribution to complex-trait susceptibility. His results suggested that although epigenetic changes can add to individual disease risk, they do not necessarily contribute to heritability, unless the stability of methylation transmission during meiosis is high [63]. In plants, transgenerational studies show that transmission of methylation patterns is stable over up to eight generations [50], but such data are not yet available for humans. Estimating the epigenetic contribution to phenotype heritability will depend on assumptions made regarding the stability of methylation transmission during meiosis, age-, tissue- and population-specific proportions of methylation changes, the contribution of methylation changes to disease risk, and the suitability of the multiplicative model. More direct data are needed to test these underlying assumptions and therefore establish whether epigenetics contributes to the missing heritability. Significant epigenetic contributions to complex phenotypes can also explain the 30-year-old paradox [67] that, in laboratory bred isogenic mice, artificially created monozygotic twins (from the splitting of a single egg) show a greater degree of phenotypic similarity than dizygotic twins (from two fertilized eggs), despite both groups being apparently identical genetically and housed in controlled environments [68].

Despite the evidence that DNA methylation is heritable, substantial changes in methylation patterns can take place over time [37–41,69], suggesting that certain regions of the genome are either undergoing epigenetic drift, or perhaps contribute to the aging process. It is therefore important to obtain estimates for the timing of epigenetic changes and how long these persist in mitotic transmissions in different human tissues. Longitudinal studies [38,49,69] imply that the precise extent of methylation can vary considerably over the scale of years, however, time-related changes in methylation tend to be modest at sites that are either completely methylated or unmethylated. Furthermore, time-related changes in methylation need to be identified with respect to disease onset and progression so as to distinguish between epigenetic changes that could be causal and those that arise secondary to disease. The change in methylation patterns with age suggests that epigenetic heritability can be thought of as a dynamic process, whereby a combination of permanent genetic effects reflecting the identity of the primary DNA sequence, cumulative stochastic changes occurring at each mitosis, and temporary environmental effects and insults can trigger epigenetic changes, and epigenetic heritability in a specific genomic region can decrease with time.

## The discordant MZ twin model and epigenetics

Phenotype differences between MZ twins reared apart are not significantly higher than between MZ twins reared together [70]. Rates of disease discordance in MZ twins are usually well over 50%, even for highly heritable disease [71–73], suggesting that epigenetics can contribute significantly to MZ twin phenotype discordance [56,74]. Discordant MZ twins have been identified in a number of diseases with rates of discordance increasing inversely with disease prevalence, where discordance is calculated as a function of prevalence. For example, in rheumatoid arthritis (RA) and schizophrenia MZ discordance rates are around 80% and prevalence rates are around 1% [72,75], whereas MZ discordance in osteoarthritis is around 40% and prevalence is around 20% [76]. Over the past two decades the discordant MZ twin design has emerged as a powerful tool for detecting phenotype risk factors while controlling for unknown confounders. Successful discoveries, which had been difficult to achieve from conventional observational epidemiology, include the influence of smoking and alcohol use on bone, the effects of social class and exercise on aging, and the causality of C-reactive protein in heart disease and obesity [77–80]. The discordant twin model is therefore helpful in resolving complex epidemiological questions and in detecting risks of small individual effect with samples as small as 20–50 twin pairs.

Recently, several epigenetic studies of MZ discordant twins have examined differences in DNA methylation profiles, aiming to identify differentially methylated regions (DMRs) in human disease [81–85]. One of the earliest studies examined methylation in the dopamine D2 receptor gene (*DRD2*) in two discordant or concordant pairs of schizophrenic twins; this study found greater methylation differences between discordant MZ twins than in unrelated cases [85]. Two subsequent studies of bipolar disorder [83] and caudal duplication anomaly [84] also identified some phenotype-associated methylation changes in discordant MZ twins, whereas a third study reported variation in methylation in the catechol-O-methyltransferase gene in birth-weight discordant MZ twins [86]. In addition to these studies, DNA methylation at single imprinted regions has also been found to differ between MZ twins discordant for Beckwith–Wiedemann syndrome [87]. All these studies examined relatively small numbers of twins with relatively low levels of epigenetic coverage. A recent study of autoimmune disease in 15 pairs of twins with systemic lupus erythematosus (SLE), RA, and dermatomyositis assayed genome-wide methylation profiles [82]. This study found 49 significant DMRs in SLE involving immune-system-related genes, but no differences were observed in RA and dermatomyositis [82]. The most recent discordant twin study used a genome-wide approach using RRBS (Box 1) in CD4+ cells from three twin pairs [81] and reported no clear differences between twins discordant for multiple sclerosis (MS). However, only three twin pairs were included in the analysis, and these comprised a heterogeneous mix of males, females, Europeans and African-Americans. Although these findings could represent true negative results, or tissue-, age- or disease-heterogeneity, it is also probable that the sample size was too small to provide statistical power to detect significant epigenetic differences. This was also the first study to use next-generation sequencing technologies to assay methylation in disease-discordant twins. Further studies should validate these results with larger case numbers and identify a similar effect in other autoimmune diseases.

The power of discordant MZ twin studies to detect DMRs will depend on a number of factors, including the effect size of the epigenetic change on the phenotype, the similarity of methylation profiles between MZ twins, sample size, and the sensitivity and coverage of the methylation assay. An estimate of the power of the discordant MZ twin design for a specific microarray methylation assay [45] found that a relatively small number (15–25) of phenotypically discordant twin pairs had sufficient (>80%) power to detect epigenetic changes of 1.2-fold, where an effect size of 1.2-fold change was significantly greater than the null experimental variance threshold for the assay (1.15-fold change) [88]. However, these power estimates do not necessarily apply to other genome-wide methylation assays, for example MeDIP-seq or Bi-seq (Box 1), which differ in sensitivity, specificity, and coverage. Therefore, the issue of power to detect epigenetic changes in the discordant MZ design needs to be revisited in view of next-generation technologies.

## Future directions in epigenetic studies on twins

There are several aspects of epigenetic studies where twins present novel opportunities to understand the biology and the mechanisms underlying complex traits. We suggest a few examples.

### Unraveling phenotypic complexity

Twin resources such as the MuTHER (multiple tissue human expression resource) project (http://www.muther.ac.uk/), which aims to assay gene expression variation in multiple tissues in twins and to identify regulatory genetic variants, can be linked with epigenetic data to explore the tissue specificity and functional consequences of epigenetic variation in twins. This project is being extended to RNA sequencing (via the EUroBATS project), and this will also allow differential allelic expression to be explored. Allele-specific expression (ASE) patterns are relatively common and are under strict genetic control [89]. ASE patterns are of interest because they are often under allele-specific methylation (ASM) control: they are typically observed in X-chromosome inactivation and in imprinted regions, but can also occur in non-imprinted autosomal genes. ASM is widespread throughout the genome [90,91] and is of interest in twin studies where a spectrum of ASM is likely to be observed and could be partitioned into epigenetic heritability and phenotype contribution. The power of combining multiple types of biological data in normal and phenotype-discordant twins will allow us to address the pleiotropic effects of genetic and epigenetic changes, that is, changes affecting multiple phenotypes, and help the interpretation of GWA studies and potentially provide insight into evolutionary mechanisms.

### Are epigenetic changes causal or secondary to the phenotype?

The timing of the epigenetic changes is crucial to understanding their role in complex traits. There is a need to measure the ‘baseline’ epigenetic profile in multiple tissues before disease onset, ideally at birth or at the beginning of adulthood, with sampling at regular intervals thereafter. There are ambitious ongoing efforts to obtain newborn-twin epigenetic profiles as part of longitudinal studies [43,92,93]. DNA methylation analysis of multiple tissues from newborn twins reveals both genetic and intrauterine components to variation in the human neonatal epigenome [43]. These data will also help us understand the effects of the intrauterine environment on epigenetics.

### In vitro fertilization (IVF) twins and epigenetics

Epigenetic profiling at birth in twins is also relevant to determining whether assisted reproduction technologies (ARTs) affect epigenetics. ARTs include IVF and related technologies [94] and have been linked to an increase in multiple births and low birth weight. About a third of all European twins are now born as a result of ART. It is possible that epigenetic marks are introduced as a result of perturbations in the intrauterine environment associated with ART, and these could affect early development. Furthermore, birth weight (which has both genetic and environmental influences and is governed by imprinted genes) is controlled at least in part by epigenetic factors [95,96]. This could be the mechanism for the Barker or fetal origins hypothesis that fetal undernutrition in middle-to-late gestation leads to disproportionate fetal growth, and can program later coronary heart disease [97]. However, to date there is no evidence linking multiple human births following ART with abnormal epigenetic modifications. To address the potential role of epigenetics in ART and separate an inherited (infertility) modification from a secondary one due to ART, comparisons of epigenetic profiles in non-ART and ART twins across different ages could be performed [98].

### Ongoing large-scale epigenome projects in twins

A recent large-scale study (EpiTwin – http://www.twinsuk.ac.uk/) aims to discover methylated genes responsible for discordance of ten common traits and diseases. The study is using MeDIP-seq on blood samples to assay epigenomic differences in 5000 adult UK twins aged 16–85, discordant and concordant for a wide variety of diseases and environments. Next-generation sequencing, although currently at significant cost, has the potential to prove powerful in detecting disease-related methylation differences at a high level of resolution in a sample of this size. Another ongoing large-scale prospective study consists of a cohort of Australian newborn twins [43,92]. These data will prove invaluable to unraveling the timing of methylation changes over the lifetime of an individual. A Norwegian study is exploring healthy twins for DNA methylation and histone-modification pattern variability across the genome, and initial findings showed relatively low epigenetic heritability at the major histocompatibility locus [99,100]. Differences in DNA methylation using array-based technologies are also currently underway in major psychosis [101] and autism [102]. Lastly, another ongoing project that presents perhaps a more cost-effective approach to next-generation epigenomic studies is that undertaken by the ENGAGE consortium (http://www.euengage.org/), where MeCAP-seq is being performed by sample pooling across multiple traits in discordant twins.

### Forensics and tissue transplantation

Two further areas that can benefit from the epigenetic differences observed in MZ twins are forensic science and medical transplantation. Although twins are no more likely to be criminals than the general population, one in 250 people are MZ twins and legal cases involving MZ twins are high-profile. For example, the genetic identity of MZ twins can allow twins to provide each other with alibis in criminal cases. Differences in epigenetic profiles between MZ twins, if consistently replicated, could in future lead to closing this loophole. In transplantation there are reports of occasional graft failures in identical twins. Studying subtle differences in twin epigenetic profiles could improve transplantation outcomes, where small epigenetic changes of immune-related genes in the host or in transplanted organs could affect transplant success [103]. Again, having a baseline of ‘normal’ epigenetic differences between MZ twins at different ages could guide the evaluation of epigenetic alterations relevant to transplantation.

## Concluding remarks

The study of epigenetic profiles in twins offers an excellent opportunity to understand the causes and consequences of epigenetic variation. Twin epigenetic heritability estimates tell us about the genetic control of DNA methylation variability and the stability of methylation patterns during cell division. The contribution of epigenetic variants to complex phenotypes can be assessed using disease-discordant MZ twins who are otherwise matched for genetics, age, sex, cohort effects, maternal effects and a common environment. These twin designs are considerably more powerful discovery tools than studies on singletons. In the near future, large-scale epigenetic studies in twins across different ages, tissues, and diseases will improve our understanding of the etiology and mechanisms of a wide range of common complex traits and diseases.

## Box 1 Next-generation sequencing technologies applied to epigenetics

The availability of next-generation sequencing technologies has recently allowed the survey of genome-wide epigenetic variation at high resolution [8,99,104,105]. We describe some of these approaches briefly below. In addition, single-molecule sequencing technologies (e.g. [106]) will probably reveal an even more complex and diverse layer of epigenetic mechanisms and modifications.

### DNA methylation

(i) Bisulfite sequencing (Bi-seq) and reduced-representation Bi-seq (RRBS). These methods are based on bisulfite conversion of the DNA followed by sequencing. During bisulfite conversion, unmethylated cytosines are converted to uracils, whereas methylated cytosines remain unchanged, therefore complete bisulfite conversion is crucial to identifying correctly the unmethylated fraction of the genome. Bisulfite treatment cannot distinguish between methylated cytosines and 5-hydroxymethylated cytosines. Bisulfite-sequencing approaches survey DNA methylation across the entire genome (Bi-seq) or in a fraction of the genome (RRBS), which tends to be enriched for clusters of CpG dinucleotides.
(ii) Methylated DNA immunoprecipitation sequencing (MeDIP-seq), methylated DNA capture by affinity purification sequencing (MeCAP-seq), and methylated DNA binding domain sequencing (MBD-seq). These techniques are based on extraction of the methylated sections of the genome by antibody or protein binding, followed by immunoprecipitation and sequencing. In some of these technologies the CpG density of a DNA fragment will affect precipitation, but algorithms that take this into account have been developed [9]. One advantage of the MeDIP-seq approach is the option to design antibodies specific to methylated cytosines, thereby allowing the separate detection of methylated cytosines and 5-hydroxymethylated cytosines.
(iii) Methylation-sensitive restriction enzyme sequencing (MRE-seq). This approach detects typically unmethylated DNA and involves DNA digestion with methyl-sensitive restriction enzymes, such as *Hpa*II, *Hin*6I and *Aci*I, to select unmethylated DNA restriction enzyme cut-sites, followed by sequencing.

### Histone modification

Chromatin immunoprecipitation followed by sequencing (ChIP-seq) is the standard approach used to detect histone-tail modifications.

### Non-coding RNAs

Long non-coding RNAs can be detected using RNA-sequencing studies by selecting RNA transcripts that are not translated and are absent from protein databases, although the absence of protein products needs to be experimentally verified. Small non-coding RNAs, including miRNAs, typically require a separate library protocol and RNA sequencing experiment.

### Chromatin structure assays

Recent technologies have been designed to assay chromatin structure directly, and include DNase-seq, in which DNase I hypersensitivity (HS) sites are sequenced [107]; FAIRE-seq, or formaldehyde-assisted isolation of regulatory elements followed by sequencing, in which nucleosome-depleted DNA is isolated from human chromatin [108]; sono-seq, where sonication of cross-linked chromatin is followed by sequencing [109]; 3C-seq, or chromosome conformation capture sequencing and related approaches [110–113]; and nucleosome positioning assays.

## Box 2 Estimating genetic heritability using twin studies

Heritability refers to the proportion of the phenotypic variance in the population that is attributed to genetic variation, where genetic variants are represented either by additive genetic values (for narrow-sense heritability estimates, h^2^) or total genetic values (for broad-sense heritability estimates, H^2^). Twin heritability estimates usually refer to the narrow-sense heritability (h^2^), which is the proportion of the total phenotypic variance in the population that is attributable to additive genetic effects. Twin estimates of heritability compare phenotypic similarities in MZ and DZ twins, because MZ twins are genetically identical and share 100% of their germline sequence variation, whereas DZ twins are not genetically identical and share only on average 50% of germline sequence variants. In addition, MZ and DZ twins share the same uterus and birth date, and are exposed to very similar environmental factors in early development. In twins, h^2^ is typically calculated as twice the difference between MZ and DZ correlations,

$$h^{2}=2(r_{MZ}-r_{DZ})$$

where *r*_*MZ*_ and *r*_*DZ*_ are the correlations of the trait in MZ and DZ twins respectively. The advantage of the classical twin study is that it is possible to estimate and distinguish between the contributions of genetic, shared environmental, and unique environmental components to the phenotype. Heritability estimates are population-specific and, unless environmental factors remain constant, environment-specific. Interpreting heritability estimates should avoid common misconceptions [34]; for example, a large estimate of heritability does not necessarily relate to underlying genetic variants of large effects, and a small estimate of heritability does not necessarily imply low additive genetic variance (for in-depth reviews of twin heritability refer to [33,34]).
